# Galectin-3 Involvement in Cognitive Processes for New Therapeutic Considerations

**DOI:** 10.3389/fncel.2022.923811

**Published:** 2022-07-05

**Authors:** Nataša R. Mijailović, Katarina Vesic, Dragana Arsenijevic, Maja Milojević-Rakić, Milica M. Borovcanin

**Affiliations:** ^1^Department of Pharmacy, Faculty of Medical Sciences, University of Kragujevac, Kragujevac, Serbia; ^2^Department of Neurology, Faculty of Medical Sciences, University of Kragujevac, Kragujevac, Serbia; ^3^Center for Molecular Medicine and Stem Cell Research, Faculty of Medical Sciences, University of Kragujevac, Kragujevac, Serbia; ^4^University of Belgrade-Faculty of Physical Chemistry, Belgrade, Serbia; ^5^Department of Psychiatry, Faculty of Medical Sciences, University of Kragujevac, Kragujevac, Serbia

**Keywords:** galectin-3, cognition, neuroinflammation, neurodegeneration, galectin-3 inhibition, microglia

## Abstract

Cognitive impairment may be a consequence of the normal aging process, but it may also be the hallmark of various neurodegenerative and psychiatric diseases. Early identification of individuals at particular risk for cognitive decline is critical, as it is imperative to maintain a cognitive reserve in these neuropsychiatric entities. In recent years, galectin-3 (Gal-3), a member of the galectin family, has received considerable attention with respect to aspects of neuroinflammation and neurodegeneration. The mechanisms behind the putative relationship between Gal-3 and cognitive impairment are not yet clear. Intrigued by this versatile molecule and its unique modular architecture, the latest data on this relationship are presented here. This mini-review summarizes recent findings on the mechanisms by which Gal-3 affects cognitive functioning in both animal and human models. Particular emphasis is placed on the role of Gal-3 in modulating the inflammatory response as a fine-tuner of microglia morphology and phenotype. A review of recent literature on the utility of Gal-3 as a biomarker is provided, and approaches to strategically exploit Gal-3 activities with therapeutic intentions in neuropsychiatric diseases are outlined.

## Introduction

Cognition as a higher brain function includes major cognitive domains such as memory, attention, language, executive and visuospatial functions. Various diseases, somatic disturbances or toxins can affect all these domains and lead to cognitive dysfunction. Cognitive impairment could be a consequence of chronic neurodegenerative processes characterized by poor learning and memory (Nilsson, 2006), which can lead to severe personality deterioration (Caselli et al., 2018). Cognitive functioning should be interpreted not only in the context of physiological aging and the onset of dementia (Popa-Wagner et al., 2015), but also as a hallmark of neurodegenerative (Chen et al., 2018) and psychiatric diseases (Millan et al., 2012), stroke (Rist et al., 2013), traumatic brain injury (Christensen et al., 2008), etc.

In physiological and pathological conditions, early identification of individuals at risk of cognitive decline is critical in neuropsychiatric clinical practice, as is maintenance of cognitive potential. There is an urgent need for biomarker discovery and validation as a clinical tool for early detection, prognosis, monitoring of cognitive alterations, and development of strategies to preserve cognitive abilities.

In exploring the molecular concepts underlying cognitive changes in neuropsychiatric diseases, galectin-3 (Gal-3), a member of the lectin family, has received considerable attention (Dumic et al., 2006). The interactions of Gal-3 with glycoproteins/glycolipids modulate cellular responses that are now research targets in the context of neuroinflammatory and neurodegenerative processes (Rahimian et al., 2021a), with the goal of developing novel therapeutics. Since Gal-3 is secreted to the cell surface and into biological fluids such as serum and urine, but also released from injured and inflammatory cells, it can be used as a valuable biomarker (Dong et al., 2017). This lectin may play either a complementary or a contrasting role in the central nervous system (CNS) and its function, due to its context-dependent activities (Shin, 2013). Although the involvement of Gal-3 in cognitive functioning has been partially investigated, comprehensive links between Gal-3 and cognitive impairment have yet to be discovered. Neuroinflammation is known to be in the background of neurological (Mishra et al., 2021) and psychiatric disorders (Bauer and Teixeira, 2019). Intrigued by this versatile molecule, we reviewed the recent neuroinflammatory/neurodegenerative properties of Gal-3 in the context of changes in cognitive functioning, as well as its immunomodulatory potential in fine-tuning microglia morphology and phenotype. The overview of Gal-3 activities and binding preferences presented here will aid in the development of novel pharmaceutical treatments targeting cognition.

## Galectin-3 Allocation and Brain Function

### Galectin-3 Main Features

Galectin-3 is a pleiotropic protein of the 15-member lectin family that is characterized by galactose-binding domains and is widely expressed in various cell types (Yang et al., 2008), in macrophages, natural killer cells, T and B cells, neutrophils and eosinophils, and is involved in the immune response (Sato et al., 2009).

Galectin-3 contains an N-terminal peptide and a C-terminal carbohydrate recognition domain and is distinguished by its unique chimeric association. This self-organization is especially important as it reflects specific intermolecular forces that govern its chemical behavior and interactions and enable efficient inhibitory pathways.

Another important determinant of Gal-3 interactions depends on its localization. In cells, it is found in both the cytosol and nucleus, but is also secreted into the extracellular space (Yang et al., 2008), where it interacts with glycoproteins that serve as an extracellular matrix for cell adhesion (Ochieng et al., 2002) and regulates apoptosis (Nangia-Makker et al., 2007). Intracellular Gal-3 interacts with other proteins and activates intracellular signaling pathways (Jeon et al., 2010; Melo et al., 2011) that trigger various physiological responses, including cell activation (Liu et al., 2011), as well as pathological conditions, such as acute and chronic inflammation (Liu and Rabinovich, 2010). All these processes have an important impact on neurodegeneration (Soares et al., 2021).

### Galectin-3 Brain Expression

Galectin-3 is expressed in a variety of glial cells in CNS tissues, including microglia, astrocytes, and oligodendrocytes (Pasquini et al., 2011), and contributes to neuroblast migration during physiological brain development (Comte et al., 2011) and differentiation (Pasquini et al., 2011). However, glial cells are not the only Gal-3 allocated scaffolds, as there is also evidence for Gal-3 expression in neurons as confirmed by the neurochemical profiles of adult normal rat brains (Yoo et al., 2017). The additional feature of Gal-3 in neurons in the CNS is that it can stimulate neuronal cell adhesion and neurite outgrowth (Pesheva et al., 1998). Neuronal expression of Gal-3 has been observed in several functional parts of the cerebral cortex and other subcortical nuclei in the hypothalamus and brainstem (Yoo et al., 2017).

### Galectin-3 and Hippocampal Dysfunction

Hippocampal dysfunction can impair cognitive abilities because the hippocampus plays a critical role in memory and learning (Giovanello et al., 2009; Hitti and Siegelbaum, 2014). Neuroinflammation biomarkers are related to brain and hippocampal volume (Gu et al., 2017), another predictor of cognitive decline (Morra et al., 2009). The expression pattern reveals that Gal-3 levels are up to 6-fold higher in the hippocampus than in the frontal cortex, olfactory bulb, striatum, and amygdala, suggesting a role for Gal-3 in learning/memory (Chen et al., 2017). Negative regulation of memory formation is associated with inhibition of integrin α3-mediated signaling and phosphorylation of Gal-3 at Serine-6 (Chen et al., 2017). On the other hand, phosphorylated Gal-3 promotes axonal branching in cultured hippocampal neurons (Díez-Revuelta et al., 2010).

### Galectin-3 in Neuroinflammation and Neurodegeneration

Microglia, the resident macrophages of the CNS, are described as never-resting cells and have critical functions under physiological and pathological conditions (Sierra et al., 2014). Neuroinflammation is considered the most important mechanism in neurological dysfunctions (Boitard et al., 2014) and consequently leads to neurodegeneration (Ransohoff, 2016). Sustained microglial activation in chronic inflammation leads to the release of inflammatory cytokines such as tumor necrosis factor α (TNF-α), interleukin-6 (IL-6), and IL-1β (Muzio et al., 2021) and affects neuronal plasticity, impairs memory, and leads to tissue damage causing neurodegenerative disorders (Cai et al., 2014; Muzio et al., 2021).

Galectin-3 is considered critical for microglial activation (Rahimian et al., 2021a) and modulates the brain innate immune response by acting as an endogenous modulator of neuroinflammation/neurodegeneration (Boza-Serrano et al., 2014, 2019). It is significantly involved in the development of these conditions through cell adhesion, proliferation, migration, apoptosis, inflammation, and modulation of the adaptive immune system (Dumic et al., 2006). Rodent studies suggest that Gal-3 impairs memory through inflammation (Boza-Serrano et al., 2019; Ramírez et al., 2019).

## Galectin-3 and Cognition

### Galectin-3 and Cognition in Neurodegenerative Disorders

**FIGURE 1:**
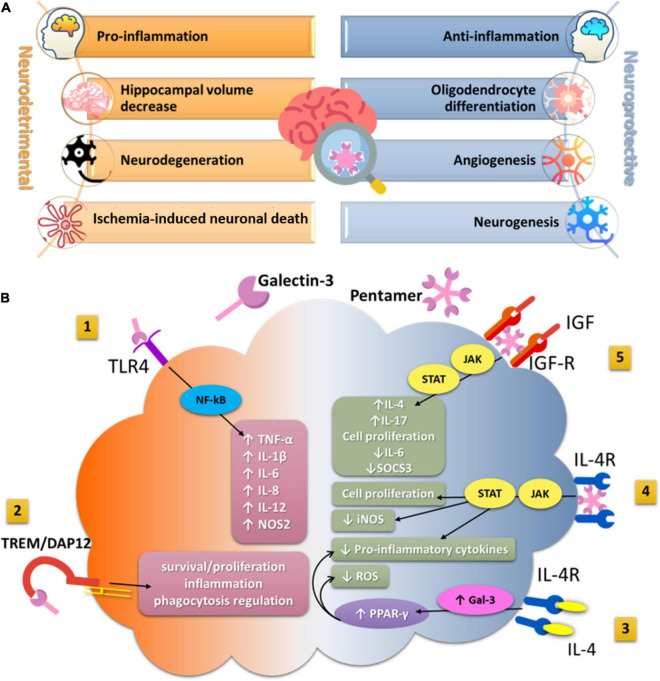
**(A)** The effects of Gal-3 in hippocampal tissue related to cognitive functioning. **(A)** Represents the summary of the main neurodetrimental and neuroprotective effects of Gal-3 in hippocampal tissue which potentially impact cognitive functioning. **(B)** The impact of Gal-3 on microglia functions in a neuroinflammatory context. (1) Secreted Gal-3 directly binds to TLR4 on the microglia surface and exacerbates inflammatory response by enhancing the production and secretion of proinflammatory cytokines and reactive nitrogen species. (2) Acting as a ligand for TREM2, Gal-3 activates TREM2/DAP12 signaling pathway and may further regulate processes of phagocytosis, inflammation, and proliferation. (3) IL-4 can interact with its tyrosine kinase receptor IL4-R on the microglia surface and increase intracellular production of Gal-3 that further activates transcriptional factor PPAR-γ. (4) The lattice formation of Gal-3 induces microglia activation through interaction with the interleukin-4 receptor (IL4R) and activates anti-inflammatory signaling. (5) By crosslinking insulin-like growth factor 1 receptor (IGFR-1), secreted Gal-3 prevents early endocytosis and over-activates the JAK/STAT signaling pathway and the transcription of genes needed for cell proliferation. TLR4, Toll-like receptor4; TREM2, triggering receptor expressed on myeloid cells-2; DAP12, DNAX-activating protein of 12 kDa; PPAR-γ, peroxisome proliferator-activated receptor gamma; IL-4, Interleukin 4; IL-4R, Interleukin-4 receptor; JAK/STAT, Janus kinase/Signal transducer and activator of transcription; iNOS, inducible nitric oxide synthase; NOS2, nitric oxide synthase-2; ROS, reactive oxygen species; IGF, insulin-like growth factor; IGF-R, insulin-like growth factor receptor; NF- κB, nuclear factor kappa-B; SOCS3, suppressors of cytokines signaling 3; TNF-α, tumor necrosis factor alpha; IL, interleukin.

Neurodegenerative diseases are debilitating and characterized by cognitive deterioration, and at the present time, these conditions are not completely curable (Feigin et al., 2019). LGALS3 genetic variation in the Gal-3 encoding gene has been associated with poorer neurocognitive function in patients with Alzheimer’s disease (AD) (Trompet et al., 2012). Serum and cerebrospinal fluid (CSF) Gal-3 levels were significantly elevated in AD patients and patients with amyotrophic lateral sclerosis (ALS) compared with healthy individuals (Ashraf and Baeesa, 2018). Elevated Gal-3 levels correlated with significant loss of memory and cognitive skills in the AD group, while this effect was not observed in ALS patients. In AD patients, serum Gal-3 levels correlate significantly with the severity of memory loss and disease stage (Wang et al., 2015; Yazar et al., 2021). Overexpressed Gal-3 in the brain and CSF may alter amyloid plaque aggregation and increase plaque-associated toxicity in AD patients (Cai et al., 2014; Ozben and Ozben, 2019; Tan et al., 2021). Gal-3 was strongly upregulated in the brains of AD patients and 5x familial AD (FAD) transgenic mouse model of AD and specifically expressed in Aβ plaque-associated microglia (Boza-Serrano et al., 2019). In Gal-3 knockout 5xFAD and APP/PS1 mice, microglia-associated immune responses were attenuated, amyloid plaques were reduced, and cognitive behavior was improved (Boza-Serrano et al., 2019; Tao et al., 2020). Furthermore, the expression of disease-associated microglia markers such as CD45, CD68, Clec7a, and proinflammatory cytokines such as TNF-α, IL-6, IL-8, and IL-12 was downregulated in the 5 × FAD/Gal3KO mice (Boza-Serrano et al., 2019). Gal-3 also acts as a ligand of toll-like receptor 4 (TLR4), which is one of the canonical receptors involved in the microglial inflammatory response (Burguillos et al., 2015). The results of this study demonstrated that added Gal-3 activated triggering receptor expressed on myeloid cells 2 (TREM2)–DAP12-dependent signaling in a dose-dependent manner (Boza-Serrano et al., 2019), indicating the role of Gal-3 as an endogenous TREM2 ligand, a key receptor driving microglial activation in AD, which is involved in the complex regulation of processes of phagocytosis, inflammation, and cell proliferation (Yuan et al., 2016; Gratuze et al., 2018; Figure 1B).

In addition, several studies showed that Gal-3 levels in brain and plasma were higher in experimental models and patients with Huntington’s disease (HD) (Siew et al., 2019) and ALS (Zhou et al., 2010; Yan J. et al., 2016) than in healthy controls and correlated with disease severity. Increased expression of Gal-3 in HD mice induced inflammation through nuclear factor kappa-B (NF-κB) and nucleotide-binding oligomerization domain-like receptor (NOD), leucine-rich repeat- (LRR), and pyrin domain-containing protein 3 (NLRP3) inflammasome-dependent pathways, while the silencing of Gal-3 suppressed inflammation, improved motor dysfunction, and increased survival in HD mice (Siew et al., 2019). Thus, suppression of Gal-3 ameliorates microglia-mediated pathogenesis, which suggests that Gal-3 is a novel druggable target for HD.

In patients with idiopathic Parkinson’s disease (PD), elevated serum Gal-3 levels significantly correlated with disease severity (Cengiz et al., 2019; Yazar et al., 2019). Gal-3 is involved in microglial activation through accumulation of α-synuclein and upregulation of proinflammatory cytokines, triggering loss of dopamine neurons. Inhibition of Gal-3 activity with inhibitory molecule (bis-(3-deoxy-3-(3-fluorophenyl-1H-1,2,3-triazole-1-yl)β-D-galactopyranosyl)-sulfane) and using knockout mice led to a significant reduction in the α-synuclein-induced inflammatory response (Boza-Serrano et al., 2014). These results suggest that genetic downregulation or pharmacological inhibition of Gal-3 might also be a novel therapeutic target in PD.

Experiments with Gal-3-deficient mice suggest that Gal-3 plays an important pathogenic role in experimental autoimmune encephalomyelitis (EAE), the animal model of multiple sclerosis (MS). Gal-3 prevents cell apoptosis by increasing proinflammatory and decreasing anti-inflammatory cytokines (Jiang et al., 2009). Similar results were observed in virus-induced MS models, where inhibition of Gal-3 decreased immune cell numbers and chemokine expression and restored cell proliferation in the subventricular zone (James et al., 2016).

These studies demonstrate that Gal-3 plays an important role in promoting the proinflammatory response in neurodegenerative disorders and contributes to cognitive decline. Furthermore, these findings indicate that inhibition of Gal-3 attenuates the proinflammatory phenotype in microglia, suggesting that Gal-3 inhibitors may have a potential therapeutic effect in these disorders.

In contrast, Gal-3’s positive influence on oligodendrocyte differentiation has been reported in brain tissue with MS (Thomas and Pasquini, 2018, 2019). Gal-3 has been shown to be an important mediator of microglia-oligodendrocyte crosstalk by binding glycoconjugates present on oligodendrocyte progenitor cells. Extracellular Gal-3 drives early process outgrowth through enhanced actin assembly and a decrease in extracellular signal-regulated protein kinase (ERK) 1/2 activation. Later, Gal-3 induces Akt activation and increases myelin basic protein (MBP) expression, promoting the release of gelsolin and actin disassembly, and thus regulating the final differentiation of oligodendrocytes (Thomas and Pasquini, 2018). These findings may govern novel therapeutic procedures comprising Gal-3 delivery to the CNS for the treatment of demyelinating diseases.

Deletion of Gal-3 in a mouse model of ALS resulted in rapid disease progression, and increasing in microglia, TNF-α, and oxidative injury (Lerman et al., 2012), suggesting that endogenous production of Gal-3 by microglia may, at least in part, limit neuroinflammation and disease progression during ALS. Thus, not only inhibition but also production of Gal-3 may play a role in neurodegeneration and the cognitive decline that may accompany it.

### Galectin-3 and Cognition in Ischemic and Neuronal Injury

Cognitive decline following ischemic stroke (Kuźma et al., 2018) and brain injury (Gorgoraptis et al., 2019) is well documented, but the role of Gal-3 in these conditions remains to be elucidated. The incidence of post-stroke cognitive impairment (PSCI) increases with Gal-3 serum level elevation, suggesting that Gal-3 may be an independent predictor of the PSCI (Wang et al., 2021). A negative role of Gal-3 is documented in the regulation of neuronal plasticity. The absence of Gal-3 allows recovery of motor functions after spinal cord injury (Mostacada et al., 2015). Gal-3 also contributes to the hypoxic-ischemia injury in the hippocampus and striatum, particularly in male mice (Doverhag et al., 2010) and ischemia-induced neuronal death in the hippocampus (Satoh et al., 2011; Hisamatsu et al., 2016). Plasma Gal-3 levels are elevated after intracerebral hemorrhage (Yan X.J. et al., 2016), subarachnoid hemorrhage (Liu et al., 2016), and birth asphyxia in new-borns (Sävman et al., 2013), and are associated with injury severity, predicting poorer clinical outcomes. Gal-3 acts as an alarmin under conditions of brain trauma and elicits a potent proinflammatory response via activation of TLR4. Overexpression of Gal-3 in microglia and CSF was observed 24 h after head injury, whereas administration of a neutralizing antibody against Gal-3 decreased the expression of IL-1β, IL-6, TNF-α, and nitrous oxide system (NOS) 2 in cortical and hippocampal cell populations (Yip et al., 2017; Table 1).

**TABLE 1:** Brief summary of clinical studies on the level and impact of Galectin-3 on main clinical findings in various neurological disorders.

Authors, year	Disease	Sample	Galectin-3 levels	Main clinical findings
Ashraf and Baeesa, 2018	Alzheimer’s disease	Serum CSF	↑	↓CF
Ashraf and Baeesa, 2018	Amyotrophic Lateral Sclerosis	Serum CSF	↑	No impact on CF
Yazar et al., 2021	Alzheimer’s disease	Serum	↑	↓CF (↓MMSE) ↑Duration of disease
Wang et al., 2015	Alzheimer’s disease	Serum	↑	↓CF (↓MMSE)
Cengiz et al., 2019	Parkinson’s disease	Serum	↑	A good predictor for advanced-stage disease
Yazar et al., 2019	Idiopathic Parkinson’s disease	Serum	↑	↑UPDRS scores ↑duration of disease
Siew et al., 2019	Huntington’s disease	Serum	↑	↓CF (↓MMSE) ↑Disease burden Correlation with UHDRS scores
Yan J. et al., 2016	Amyotrophic Lateral Sclerosis	Serum	↑	↑Duration of disease
Zhou et al., 2010	Amyotrophic Lateral Sclerosis	CSF spinal cord tissue	↑	Disease biomarker
Wang et al., 2021	Acute ischemic stroke	Serum	↑	↓CF (↓MoCA)
Yan X.J. et al., 2016	Intracerebral hemorrhage	Serum	↑	↑Inflammation ↑Injury severity ↑Mortality
Liu et al., 2016	Subarachnoid hemorrhage	Serum	↑	↑Disease severity Poorer prognosis
Sävman et al., 2013	Birth asphyxia	CSF	↑	Severe clinical course poorer prognosis

*CSF, cerebro-spinal fluid; CF, cognitive functions; MMSE, Mini Mental State Examination; UPDRS, Unified Parkinson’s Disease Rating Scale; UHDRS, Unified Huntington’s disease Rating Scale; MoCA, Montreal Cognitive Scale.*

In contrast, the neuroprotective potential of Gal-3 was confirmed both *in vitro* and *in vivo* in a model of acute ischemic stroke (Rahimian et al., 2019b). The application of recombinant Gal-3 after stroke increased expression of Ym1, diminished iNOS expression, and lead to a significant increase of an anti-inflammatory cytokine (IL-4) and a reduction in proinflammatory cytokines (TNF-α, IL-1β, IFN-γ, and IL-17) in ipsilateral brain regions. Furthermore, the observed shift in microglia toward an anti-inflammatory profile was associated with a significant decrease in infarct size. Gal-3 administration induced microglial ramification, as quantified by filopodia length and number (Rahimian et al., 2019b), and consequently increased microglial motility. All of these effects of Gal-3 were mediated by IL-4. IL-4 tyrosine kinase receptors are expressed by microglia, and Gal-3 was shown to interact with these receptors and enhance their activities (Partridge et al., 2004). It has been shown that endogenous IL-4 can thus lead to an increase in intracellular Gal-3 and subsequently induce the canonical transcription factor peroxisome proliferator-activated receptor (PPAR-γ) (Rahimian et al., 2019a), which decreases the production of reactive oxygen species and proinflammatory cytokines, and suppresses the activity of NF-κB (Baek et al., 2015; Figure 1B).

The important factor in the Gal-3-induced microglia proliferation and alternative activation is insulin-like growth factor 1 (IGF-1). IGF-1 has been associated with post-stroke recovery and neuroprotection by reducing brain damage in diverse experimental settings, inducing neurogenesis, and accelerating neural survival (Sohrabji and Williams, 2013). The interaction of Gal-3 with N-linked glycans attached to insulin-like GF1 receptor (IGFR1), enhances GF-induced signal transduction and cellular growth (Partridge et al., 2004). Results obtained in primary microglia cultures showed that extracellular Gal-3 is responsible for increased microglia ramifications and interaction of Gal-3 with glycosylated GF such as IGF-1 (Rahimian et al., 2018). After binding to glycans attached to GFRs, oligomerized Gal-3 molecules crosslink GFRs at the surface and delay their removal by endocytosis, resulting in prolongation of GF signaling (Partridge et al., 2004; Lalancette-Hebert et al., 2012). Gal-3 deficiency leads to the insufficient microglia activation and defective IGF-R1 signaling/mitogenic response, overexpression of IL-6 and suppressors of cytokines signaling 3 (SOCS3), suggesting the involvement of the JAK/STAT3 signaling pathway (Lalancette-Hebert et al., 2012). In addition, the presence of Gal-3 is required for early injury-induced microglia activation and induction of TLR2 response (Lalancette-Hebert et al., 2012; Figure 1B).

Biological sex and aging represent an important factor influencing the immune response by triggering various inflammatory events in stroke, as well as in post-stroke recovery (Banerjee and McCullough, 2022). A trend in greater cognitive decline and central neuroinflammation was observed in aged male mice of the stroke model (Ahnstedt et al., 2020). Rahimian et al. (2019a,2020) demonstrated that Gal-3 mediates neuroinflammation on microglia in a sex-dependent manner. Administration of glucosamine, a pharmacological Gal-3 modulator, 72 h after ischemic injury increased levels of Gal-3 and anti-inflammatory cytokines such as IL-4, IL-13, and colony-stimulating factors in male mice. These changes were followed by a marked decrease in the size of the ischemic lesion. In contrast, Gal-3 levels were decreased in female mice treated with glucosamine, which in turn was associated with increased levels of proinflammatory cytokines and larger infarction size (Rahimian et al., 2020).

Galectin-3 also exerts remodeling functions by promoting angiogenesis and neurogenesis in endothelial and neural progenitor cells and enhancing micro vessel density in ischemic rat brains (Yan et al., 2009). Gal-3 acts by promoting cellular survival and angiogenic and migratory potential under ischemic conditions *in vitro* by modulating integrin-linked kinase (ILK) signaling, whereas silencing of ILK diminishes angiogenesis and microglia migration (Wesley et al., 2013). Intracerebral local delivery of the Gal-3 into the rat brain exerted neuroprotective effects by decreasing ischemic lesion volume and neuronal cell death (Wesley et al., 2021). Gal-3 also modulates vascular endothelial growth factor- and basic fibroblast growth factor-angiogenic response, through αvβ3 integrin signaling (Markowska et al., 2010). Since it was demonstrated that microglia promptly shift towards the injured site and limit brain damage after stroke (Davalos et al., 2005; Eyo and Dailey, 2012), Gal-3 likely promotes migration of microglia, as its chemotactic effects were confirmed (Nangia-Makker et al., 2000). These results indicate a key role of Gal-3 in neurovascular protection and functional recovery following ischemic stroke through modulation of angiogenic pathways (Figure 1A).

In summary, Gal-3 can act as a protective or detrimental molecule and these effects may be time-, context-, model-, and even sex-dependent under ischemic conditions. All this complexity makes Gal-3 an interesting yet challenging druggable target in the modulation of post-stroke angiogenesis, neurogenesis, and accompanying neuroinflammation.

### Galectin-3 and Cognition in Mental Disorders

Cognitive impairment is not exclusively observed in aging, dementia, or representative neurodegenerative disorders, but also in specific aspects of cognitive functioning in mental disorders. Cognitive changes have been observed in patients with depression (Culpepper et al., 2017) along with the volume reduction in specific brain regions observed in patients with mild cognitive impairment and depression (Zacková et al., 2021). Cognitive dysfunctionalities are observed in the widespan of mental disorders: bipolar disorder (Solé et al., 2017), schizophrenia (Daban et al., 2006), autism, and hyperkinetic syndrome (Thapar et al., 2017).

Galectin-3 has been investigated in animal and human studies of different mental disorders, but mainly as an alarmin, a biomarker of a particular disease stage. There is no complete investigation of its involvement in other underlying molecular interactions leading to unique cognitive changes, which might be specific to a single mental disorder. No correlation of serum or CSF levels with clinical parameters of cognitive functioning has been established so far in primary psychiatric disorders. In patients with schizophrenia, lower serum levels of Gal-3 were measured in patients with first-episode psychosis and schizophrenia in relapse, whereas they were higher in patients with schizophrenia in remission, compared with levels determined in healthy controls (Borovcanin et al., 2018). Gal-3 has been considered in neurodevelopment, neuroinflammation, and neurodegeneration, also regarding its interplay with neurotransmitters (Borovcanin et al., 2021a), as well as its possible modulation by antipsychotic treatment and consequent obesity and cognitive changes in schizophrenia (Borovcanin et al., 2021b). Depression was independently associated with higher levels of Gal-3 in patients with type 1 diabetes mellitus (DM) (Melin et al., 2020). Gal-3 could be included in the link between obesity and depressive symptoms in overweight and obese women (Setayesh et al., 2021). According to the current state of knowledge, Gal-3 activity in cognitive functioning must be viewed from the standpoint of its role in systemic changes regulating inflammatory and metabolic processes of the whole organism.

The recent explosive growth in data regarding the homeostasis of microglia has revealed their roles in the shaping of the neural circuitry, synaptic plasticity, and phagocytosis, pointing to the importance of their functions in the contexts of cognitive control and psychiatric disorders (Rahimian et al., 2021b). Since the inflammatory CNS milieu and microglia are implicated in these disorders, the role of Gal-3 as a regulator of microglia activity is a puzzle that needs to be reconciled in future studies. Abnormal expression of TLRs, including TLR3 and TLR4, was observed in microglia, which may have important implications for multiple brain functions (Hanke and Kielian, 2011). Phagocytosis is the crucial process for the removal of apoptotic neurons and oligodendrocytes, which depends on receptors such as Gal-3, TREM2, CD11b, and T-cell membrane protein 4 (Rahimian et al., 2018). Pandey et al. (2014) reported that protein expressions of TLR2, TLR3, TLR4, TLR6, and TLR10 were significantly increased in the prefrontal cortex of depressed patients who committed suicide. Since Gal-3 acts as a ligand for TLR, this could be a possible explanation for its involvement in these diseases. In the modern treatment of autism spectrum disorders, not only BBB permeability is a hot topic, but also gastrointestinal immune barrier (Yousefi et al., 2022), which could be controlled by TLR2 and TLR4 or Gal-3 (Beukema et al., 2020).

## Galectin-3 Targeted Strategies for Preserving Cognition

The structure of Gal-3 enables versatile interactions with cell surface carbohydrates, ranging from hydrogen bonding to hydrophobic interactions (Chan et al., 2018). Many glycoproteins comprise a specific glycan epitope, but with a limited number of physiological receptors for a living cell. The processes involving Gal-3 *in vivo* are complex, therefore fine-tuning in which interactions are possible to observe come down to versatile *in vitro* methods. Sophisticated analytical methods need to be applied, both from an instrumental perspective considering sensitivity and selectivity, and from a theoretical modeling/molecular simulations perspective (Heine et al., 2022). The refinement of perspective galectin antagonists needs to incorporate detailed insight into molecular interactions supported by various instrumental methods.

The investigation for new anti-inflammatory agents has become a target of profound research, as currently available therapies fail to stop or delay progressive neuronal loss. The development of several distinct Gal-3 inhibitors may have a positive effect on disease processes (Stegmayr et al., 2019). One of the reasons is that galectins are expressed at low levels under normal physiological conditions but are markedly increased in diseases (Klyosov and Traber, 2012). Hence, a strategy to inhibit galectin expression or function may be effective without affecting normal basal function. The efficacy of galectin blockers is difficult to extrapolate from animals to humans, and these data are lacking in the literature. In addition, administration and bioavailability affect the behavior of the drug in the human body. The galectins have highly conserved residues, and the development of selective inhibitors is a major challenge. Inhibitors should have high affinity, specificity, and chemical stability in the biological environment (Stegmayr et al., 2019). Also, there are other problems such as expensive synthesis and unfavorable pharmacokinetics (Sethi et al., 2021).

Targeted therapeutics are still in the early stages of development, and there are limited data on the use of pharmacological galectin inhibitors in human diseases. Inhibitors are generally classified into carbohydrate and peptidic non-carbohydrate types (Sethi et al., 2021). Carbohydrate types include small organic molecules and galactose derivatives (Klyosov and Traber, 2012). Among small organic molecules, Gal-3 inhibitors are the most potent with nanomolar K_d_—29 nM (3,3′-ditriazolyl thiodigalactoside), 50 nM (3,3′-diamido thiodigalactoside), 320 nM (3′-amido lacNAC derivative), and 660 nM (3′-triazolyl lacNac derivative) (Klyosov and Traber, 2012; Blanchard et al., 2014). Non-carbohydrate inhibitors include peptidomimetics, peptide-based inhibitors, and heterocyclic compounds that show promising activity against Gal-3 (Mayo, 2012).

Considering the fact that hepatic encephalopathy and depression share the same properties in terms of cognitive dysfunctionality (Kronsten and Shawcross, 2022), the translational approach could be very useful in implementing knowledge about the same underlying mechanisms. The therapeutic potential of Gal-3 inhibitors has been confirmed in liver fibrosis, non-alcoholic steatohepatitis, cirrhosis, and idiopathic pulmonary fibrosis (Sethi et al., 2021). Fruit-derived pectins such as GCS-100, Davanat, Belapectin, and Modified citrus pectin (MCP) are some of the inhibitors that have been tested in clinical trials (Girard and Magnani, 2018). MCP is a polysaccharide extracted from citrus that binds to Gal-3 and acts like its antagonist (Eliaz and Raz, 2019). MCP prevents disruption of the blood-brain barrier (BBB) and brain injury in a mouse model of subarachnoid hemorrhage, indicating that Gal-3 regulates the inflammatory response in this brain injury (Nishikawa et al., 2018). People with DM type 2 are at increased risk for cognitive decline since cerebrovascular and neurodegenerative chronic complications of the disease affect brain function (Karvani et al., 2019). Serum Gal-3 levels have been shown to be significantly higher in DM type 2 patients with mild cognitive impairment compared to controls (Ma et al., 2020). Similarly, Gal-3 levels were increased in the serum and brain of high-fat diet/streptozotocin-induced diabetic rats (Yin et al., 2020), whereas the MCP attenuated inflammation, oxidative stress, and cognitive impairment in diabetic rats and also in high glucose-stimulated BV-2 microglial cells (Yin et al., 2020). These findings indicated that Gal-3 might be a potential therapeutic target for cognitive impairment in diabetes.

TD139, a novel high-affinity and cell-permeable specific inhibitor of Gal-3, was found to ameliorate the clinical and histological manifestations in the model of experimental autoimmune uveitis in mice (Liu et al., 2022). TD139 attenuated the microglial activation and inflammatory response through TLR4/MyD88/NF-κB pathway. This inhibitor significantly reduced the expression of Gal-3, iNOS, COX2, and proinflammatory cytokines including IL-6, TNF-α, and IL-1β. In patients with secondary progressive MS, Gal-3 is already marked as a target antigen for antibodies responsible for the BBB disruption, and anti-Gal-3 antibodies are proposed as a novel diagnostic marker (Nishihara et al., 2017).

In addition to inhibition of Gal-3, other therapeutic potentials of this multifunctional lectin should also be considered. It has been demonstrated that Gal-3 can be secreted and exported from cells by an alternative secretory pathway including specific vesicles/exosomes (Nickel, 2003). Modulation of Gal-3 secretion by exosomes represents an interesting new concept and a potential therapeutic approach in neurodegenerative disorders, as numerous studies on exosomes have confirmed their role in pathophysiological mechanisms and disease development, as well as their application for therapy and targeted drug delivery in these brain pathologies (Gao et al., 2021). It has been shown that naturally occurring or engineered exosomes derived from stem cells may exert therapeutic effects in AD (Perets et al., 2019). Gal-3 derived from mesenchymal stem cells removed the aberrant forms of tau and reduced hyperphosphorylation of tau *in vitro* and *in vivo* and also ameliorated deficits in spatial learning and memory, confirming its potential therapeutic role in AD pathology and accompanying memory impairment (Lim et al., 2020).

Activated microglia prepare substrates for endocytosis by releasing sialidase (neuraminidase), which desialylates glycans on cell surfaces and debris in both microglia and neurons, facilitating Gal-3 binding as an opsonin (Nomura et al., 2017). Gal-3 and desialylation may increase in a variety of brain pathologies. Thus, inflammatory loss of neurons or synapses may potentially be blocked by inhibiting neuraminidases and Gal-3, which could be a potential treatment strategy to prevent neuroinflammation and neurodegeneration (Puigdellívol et al., 2020).

The accumulation of irreversible long-lived proteins, advanced glycation end products (AGEs), and the interaction of AGEs with cellular receptors are considered key events in the development of long-term complications of chronic diseases, such as DM, AD, and aging (Schmidt et al., 2000). Gal-3 is an important receptor for advanced glycation end products (RAGEs) on the cell surface, and it has been suggested that pharmacological inhibition of AGE receptor-mediated cell activation with specific antagonists may provide the basis for therapeutic interventions in these diseases (Pugliese et al., 2015), along with the impact of ligand glycoconjugates which influence Gal-3 binding and may modulate inflammatory responses and remyelination in neurodegenerative diseases (Nio-Kobayashi and Itabashi, 2021).

## Discussion

A convergence of preclinical and clinical data has provided bases for the potential role of Gal-3 in cognition modulation in complex pathological states and diseases. Understanding the structure-function relationships of Gal-3 is complicated by its versatile glycan-binding properties and oligomeric structure. Gal-3 can crosslink membrane glycans to initiate, amplify, attenuate, or inhibit signaling pathways that lead to cell differentiation, proliferation, or death. Current challenges of cellular environment characterization leave us without a detailed understanding of galectin-binding specificities in a cellular context. Structural motifs and targets can be separated by *in vitro* studies, but the question remains of how to translate this extensive knowledge to *in vivo* metrics.

Gal-3, denoted as a two-faced molecule, triggers the activation of microglia and affects various signaling pathways in response to pro-inflammatory stimuli, and is a key determinant in neuroinflammation/neuroprotection mechanisms by recognizing glycans and their modifications. The positive or negative effects of Gal-3 are highly dependent on different brain areas, injury conditions, and disease progression (Shin, 2013). This delicate balance dictates that inhibitory pathways must be highly specific/selective.

The complex structure of Gal-3, its time- and context-dependent activities, along with genetic and gender differences make the investigation of its therapeutic potentials and modalities a very complex and difficult task, with many questions to be addressed before further clinical trials are initiated. The role of Gal-3 as a mediator of immune responses in the damaged brain and the mechanisms Gal-3 employs to affect microglial function may serve to develop novel immunomodulatory strategies for the treatment of neuropsychiatric disorders.

Gal-3 released from activated microglia appears to function as a master regulator acting as an endogenous TLR4, TLR2, and TREM ligand. In contrast, under ischemic conditions, it may also exert proangiogenic, neurogenic, and migratory potential as well as anti-inflammatory effects. Based on the presented findings, Gal-3 plays an important and complex role as an endogenous modulator of immune response and inflammation in various pathological conditions. Targeted drug delivery to injured brain regions remains an unmet challenge, which is even more prominent in neuropsychiatric disorders because the exact pathological location is often uncertain. Although still in its infancy, proteomic studies represent a promising new approach for future investigations. Unfortunately, the data regarding the use of Gal-3 inhibitors in the treatment of neuropsychiatric disorders in clinical settings are lacking at present.

Cognitive decline could also be a consequence of Gal-3 effects in systemic circulation and occurs in parallel with various somatic comorbidities. Further research needs to consider the different roles of Gal-3 in immunometabolic disorders leading to cognitive changes. As with any biomarker, the question is whether levels in peripheral blood accurately reflect levels in brain tissue. It would be important to develop selective Gal-3 inhibitors that can cross the BBB and conduct further testing to demonstrate therapeutic efficacy in brain disease models.

In this mini-review, we wanted to briefly summarize an up-to-date rationale for selectively targeting Gal-3 in the extracellular space or in its involvement in intracellular cascades for potential cognitive improvement. Gal-3 can be modulated by multiple modifications such as phosphorylation and oligomerization that lead to multiple and/or seemingly opposing effects under various pathological conditions in CNS. We have also pointed out the high expression of Gal-3 in the hippocampus and the deleterious effects of increased Gal-3 on cognition in neurodegenerative states. A particular challenge is to potentially interfere with adult brain plasticity processes to improve cognitive performance by using Gal-3 as an endogenous modulator of neuroinflammation/neurodegeneration and ligand for TLR and TREM2 as key receptors in microglial activation and brain trauma. These points also showed promise for future strategies to treat mental disorders. A clear association between Gal-3 elevation and poorer cognitive performance was found in vascular incidents and after recovery processes. However, elevated Gal-3 levels in serum and CSF were not necessarily associated with memory loss. In addition, its anti-inflammatory properties through IL-4 secretion and neuro-vascular protection through modulation of angiogenic pathways should be considered. Gal-3 involvement in immunometabolism, including IGF, has neuroprotective properties and could also have an influence on cognition. So, not only the inhibition but also additionally induced production of this interesting molecule could be considered to prevent neurodegeneration and the cognitive decline that may accompany it.

The development of therapies to prevent and/or slow the progression of cognitive decline in the early stages of neuropsychiatric disorders is of utmost importance and includes symptomatic treatments, lifestyle modifications, and risk factor management, among others. Although Gal-3 itself is not a disease-specific marker, it has been recognized as a potential biomarker for targeting the early stages of inflammatory responses in these diseases. Further animal studies are needed for a more complete view of the complex interplay between Gal-3, microglia, and neurons. In clinical settings, the time-course analysis of Gal-3 levels in serum and CSF at different stages of these diseases should be performed along with neuropsychological assessment to reveal the link between Gal-3 expression patterns and cognitive changes.

## Author Contributions

NM presented the idea and initial structuring of this manuscript. NM, MM-R, and DA prepared the figures. All authors searched the literature, wrote, and gave some new insights and suggestions regarding specific fields of their competencies, read, discussed, accepted responsibility for, and approved the entire content of the submitted manuscript.

## Conflict of Interest

The authors declare that the research was conducted in the absence of any commercial or financial relationships that could be construed as a potential conflict of interest.

## Publisher’s Note

All claims expressed in this article are solely those of the authors and do not necessarily represent those of their affiliated organizations, or those of the publisher, the editors and the reviewers. Any product that may be evaluated in this article, or claim that may be made by its manufacturer, is not guaranteed or endorsed by the publisher.
